# Phylogenetic and Taxonomic Revision of an Enigmatic Group of Haptorian Ciliates, with Establishment of the Kentrophyllidae fam. n. (Protozoa, Ciliophora, Litostomatea, Pleurostomatida)

**DOI:** 10.1371/journal.pone.0123720

**Published:** 2015-05-06

**Authors:** Lei Wu, John C. Clamp, Zhenzhen Yi, Jiqiu Li, Xiaofeng Lin

**Affiliations:** 1 Laboratory of Protozoology, Key Laboratory of Ecology and Environment Science in Guangdong Higher Education, South China Normal University, Guangzhou, 510631, China; 2 Department of Biology, North Carolina Central University, Durham, NC, 27707, United States of America; University of Connecticut, UNITED STATES

## Abstract

Haptorian ciliates in the closely similar genera *Kentrophyllum* and *Epiphyllum* possess a unique pattern of ciliature and are distinguished from one another only by the presence or absence of cytoplasmic spines projecting from the margin of the cell. Phylogenetic analyses based on SSU rDNA sequences of three new samples from coastal habitats in China revealed that species in the two genera clustered together indiscriminately (i.e. forms of neither genus clustered into an independent clade) as a maximally supported, monophyletic clade that branches basally to all other clades in the order Pleurostomatida and is strongly divergent from other members of the family in which the genera have been placed. As a result, we propose that *Epiphyllum* be synonymized with *Kentrophyllum* and that a new family Kentrophyllidae fam. n. be established for the genus. We hypothesize that the two-sutures of *Kentrophyllum* is a plesiomorphy within the Pleurostomatida and the unique peripheral kinety might represent an autapomorphy of *Kentrophyllum*. In addition, we provide a taxonomic revision of *Kentrophyllum* including description of three new species (*K*. *bispinum* sp. n., *K*. *strumosum* sp. n., and *K*. *qingdaoense *sp. n.), redescription of *K*. *verrucosum* (Stokes, 1893) Petz et al., 1995, and three new combinations (*K*. *soliforme *(Fauré-Fremiet, 1908) comb. n., *K*. *hohuensis *(Wang and Nie, 1933) comb. n. and *K*. *shenzhenense *(Pan et al., 2010) comb. n.). The surface ultrastructure of the genus *Kentrophyllum* is recorded for the first time. And a key to all known species of *Kentrophyllum* was also suggested.

## Introduction

Haptorians are a diverse group of predatory ciliates comprising more than 1000 morphospecies [1, 2]. In recent phylogenetic studies of haptorians, a steady increase in taxon sampling and the number of gene sequences and morphological characters in analyses has led to a new evaluation of their evolutionary relationships [1, 3–9]. Members of the order Pleurostomatida are haptorians with a distinctive, laterrally compressed, flattened body that are common in a variety of habitats all over the world [2]. Their biodiversity is well-documented, with over 200 morphospecies assigned to 12 genera within two families reported from a variety of aquatic environments in Europe [2, 10–18], Asia [19–31], North America [32], and even Antarctica [33, 34].

In the pleurostomatid family Amphileptidae, the relatively small genera *Kentrophyllum* and *Epiphyllum* comprise fewer than 10 morphospecies [10–14, 19, 22, 32, 35–38], differing from other amphileptids by having a large number of somatic kineties forming sutures on both right and left sides and an extremely elongate perioral kinety at the periphery of the body [22, 27, 33]. A single morphological character (absence of spines surrounding the margin of the cell) distinguishes *Epiphyllum* from *Kentrophyllum* [22].

Until now, no SSU rDNA sequences of species of *Kentrophyllum* and only one species of *Epiphyllum* have been included in phylogenetic analyses of pleurostomatid haptorians [27]. We were able to add sequences of another species of *Epiphyllum* and two species of *Kentrophyllum*, allowing us to test the hypothesis that *Epiphyllum* and *Kentrophyllum* constitute separate genera. In addition, we were able to determine the phylogenetic position of the *Kentrophyllum*-*Epiphyllum* assemblage of species relative to other members of the family Amphileptidae.

## Materials and Methods

### Collection and preparations of samples

Samples of habitat water were collected from an intertidal zone and two different mangrove wetlands in 250-ml, wide-mouth bottles. The sampling locations are public areas, thus no specific permissions were required to collect the materials necessary for the present study. No known endangered or protected species were involved in the present study.

Specimens were isolated from subsamples of this water and cultivated in Petri dishes at room temperature (~ 25°C) using rice grains to produce growth of bacteria for food. After 3–5 days, living cells were picked from cultures and observed at magnifications of 100–1000 × with bright field and differential interference contrast microscopy. Fixed specimens were stained with protargol by the Wilbert [39] method to reveal the infraciliature. Measurements of stained specimens were made at a magnification of 1000 ×. Drawings of stained specimens were made with the help of a camera lucida. *Kentrophyllum qingdaoense* for scanning electron microscopy (SEM) is prepared according to the method described by Foissner [40] to disclose the ultrastructural, in particular, the pores of contractile vacuoles and the spines. Terminology and classification follow Lin et al. [22] and Lynn [41].

### Extraction, amplification, and sequencing of DNA

One or several cells of each species were isolated, identified by morphological examination, washed several times in sterilized habitat water to remove contaminants, and transferred into 45μl ATL buffer for extraction of genomic DNA. Extraction of genomic DNA, amplification of the gene coding for SSU rDNA by PCR, and sequencing of this gene in each species were done according to the methods of Wu et al. [29].

### Phylogenetic analyses

Sequences of SSU rDNA used in phylogenetic analyses were obtained from the GenBank database. *Metopus contortus* and *Nyctotheroides parvus* (class Armophorea) were selected as outgroup taxa. Sequences were aligned with CLUSTAL W implemented in Bioedit v.7.0 [42], which was used to remove regions of ambiguously aligned characters. The final alignment of 1,590 characters representing 39 taxa was used to construct phylogenetic trees.

A Bayesian inference (BI) analysis was done with MrBayes 3.1.2 [43] using the GTR + G + I evolutionary model indicated by MrModeltest v.2 [44]. The program was run for 1,500,000 generations, with trees sampled every 100 generations, and the first 3,750 trees (25%) discarded as burn-in. A maximum likelihood (ML) analysis was done with RAxML 8.0 [45], using the GTR+I+G model selected by MrModeltest v.2, obtained through the CIPRES Portal V 1.15 (http://www.phylo.org/index.php/portal/) using default parameters and bootstrapping with 1000 replicates. A Maximum-parsimony (MP) tree was constructed with PAUP 4.0b10 [46], using the default parameters and the reliability of the internal branches was estimated by the bootstrap method with 1000 replicates [47]. The following specialized terms are used in the Results and Discussion:

**Spine—**three types of elongate cytoplasmic structures found in species of *Kentrophyllum* (Lin et al. [22]).
**Single-spine—**slender and stiff like a seta in living cells; evenly distributed around the margin of the cell except for the oral area.
**Double-spine—**pair of closely spaced single spines projecting from the same point on the margin of the cell.
**Soft spine—**distinctly soft and flexible rather than stiff, relatively thick at the base, and tapering to a sharp point; projecting singly or in a small group of 2–3 from a marginal wart rather than directly from the margin of the cell.

### Nomenclatural Acts

The electronic edition of this article conforms to the requirements of the amended International Code of Zoological Nomenclature, and hence the new names contained herein are available under that Code from the electronic edition of this article. This published work and the nomenclatural acts it contains have been registered in ZooBank, the online registration system for the ICZN. The ZooBank LSIDs (Life Science Identifiers) can be resolved and associated in formation viewed through any standard web browser by appending the LSID to the prefix “http://zoobank.org/”. The LSID for this publication is: urn:lsid:zoobank.org:pub: 17E4EE36-70B7-40A2-A676-7D40913F6728. The electronic edition of this work was published in a journal with an ISSN, and has been archived and is available from the following digital repositories: PubMed Central, LOCKSS.

## Results and Discussion

### SSU rDNA sequences

Three SSU rDNA sequences were deposited in GenBank with the following accession numbers, length, GC content and names that reflect taxonomic conclusions derived from our phylogenetic analyses: KM025125, 1531 bp, 42.19% (*Kentrophyllum bispinum* sp. n.), KM025126, 1539 bp, 41.85% (*Kentrophyllum strumosum* sp. n.), and KM025127, 1531 bp, 42.19% (*Kentrophyllum verrucosum*).

### Phylogenetic analyses

**Fig 1:**
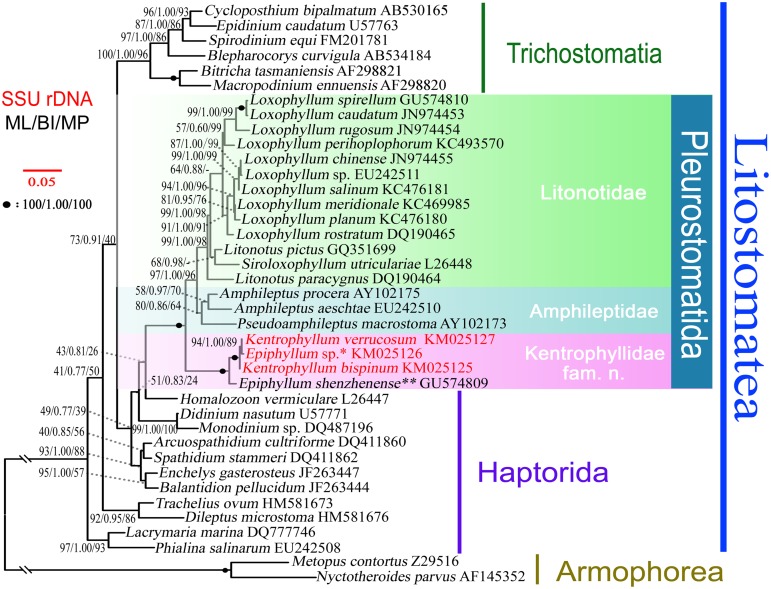
Phylogenetic tree constructed from 39 SSU rDNA sequences of litostomatean ciliates. Numbers at nodes indicate bootstrap values for maximum likelihood (ML), posterior probabilities from Bayesian analysis (BI) and bootstrap values from maximum parsimony (MP), respectively. GenBank accession numbers are given after names of species. Newly sequenced species are marked with a red. Scale bar corresponds to five substitutions per 100 nucleotide positions. * Designated as *Kentrophyllum strumosum* sp. n. in present work. ** Designated as *Kentrophyllum shenzhenense* (Pan et al., 2010) comb. n. in present work.

Trees constructed using different algorithms were essentially identical; therefore they were combined to make a single consensus tree (Fig 1). The monophyletic order Pleurostomatida (ML/BI/MP, 100/1.00/100) comprises three major clades, one of which is a maximally supported clade (ML/BI/MP, 99/1.00/98) that contains species of *Loxophyllum*, *Litonotus*, and *Siroloxophyllum* and corresponds to the family Litonotidae shown in phylogenetic analyses of previous studies [1, 3–9]. Two species of *Amphileptus* cluster with a species of *Pseudoamphileptus* with moderate support (ML/BI/MP, 80/0.86/64) form a second clade.

Species of *Kentrophyllum* and *Epiphyllum* form a third, maximally supported clade of pleurostomatids that is strongly divergent from other amphileptids, and separation of *Epiphyllum* from *Kentrophyllum* was not indicated by our molecular analyses (Fig 1). *Epiphyllum shenzhenense* was basal to a maximally supported subclade of closely similar sequences containing two species of *Kentrophyllum* and one species of *Epiphyllum*. In this subclade, *Kentrophyllum bispinum* was basal, and an association of *Epiphyllum* sp. with *K*. *verrucosum* was strongly supported (ML/BI/MP, 94/1.00/89). Therefore, the presence or absence of marginal spines is not a valid generic character. In summary, our phylogenetic analyses of the family Amphileptidae indicate that the following taxonomic changes are necessary:

- *Epiphyllum* is made a junior subjective synonym of *Kentrophyllum*.
- The family Kentrophyllidae fam. n. is proposed for *Kentrophyllum*-like species of pleurostomatid ciliates.

### Systematic treatment

#### Order Pleurostomatida Schewiakoff, 1896

**Family Kentrophyllidae fam. n.** urn:lsid:zoobank.org:act: 67599E08-B4F5-4271-9E9C-251E550C1FB6

**Type genus.** *Kentrophyllum* Petz et al., 1995

**Diagnosis.** Pleurostomatida with medial, right-hand somatic kineties gradually shortened and forming sutures both at the anterior and posterior. One peripheral dikinety on right side that completely encircles margin of cell.

**Etymology.** The new family name is derived from the name of its type genus *Kentrophyllum*.

**Comments.** Pleurostomatids are currently grouped into two families, the Litonotidae and Amphileptidae, which are separated mainly by differences in the ciliary pattern on the right side of the body (suture absent in Litonotidae vs. suture present in Amphileptidae) [2]. The genus *Kentrophyllum*, currently placed in the Amphileptidae, is characterized by having the right medial somatic kineties gradually shortened and to form two sutures, one anterior and one posterior. In the phylogenetic tree, the *Kentrophyllum* clade branches basally at the deepest level of the order Pleurostomatida. This leads us to hypothesize that the two-sutures of *Kentrophyllum* is a plesiomorphy within the Pleurostomatida. Furthermore, species of *Kentrophyllum* have one peripheral kinety (perioral kinety 2) on the right side that consists mostly of dikinetids and forms a complete circle around the margin of the cell. This feature has never been seen in any other genus of the order Pleurostomatida [2] and might represents an autapomorphy of *Kentrophyllum*.

#### Genus *Kentrophyllum* Petz et al., 1995

**Emended diagnosis.** Kentrophyllidae with a flattened laterally compressed body, with or without spines on its margin. Extrusomes evenly distributed or clustered into marginal warts.

### Revision of the genus *Kentrophyllum* emend

The genus *Kentrophyllum* was established for amphileptids with a flat body, spines along the ventral and dorsal margins, and a right perioral kinety that makes an almost complete circuit around the edge of the body [33]. Lin et al. [22] emended the original diagnosis to add formation of sutures by the somatic ciliature on the right side as a diagnostic character. To date, the following seven species of *Kentrophyllum* have been reported [22, 24, 27, 33]: *K*. *setigerum* (Quennerstedt, 1867) Petz et al., 1995; *K*. *verrucosum* (Stokes, 1893) Petz et al., 1995; *K*. *fibrillatum* (Dragesco, 1954) Petz et al., 1995; *K*. *pseudosetigerum* (Dragesco, 1954) Petz et al., 1995; *K*. *raikovi* (Dragesco, 1965) Petz et al., 1995; *K*. *antarcticum* Petz et al., 1995; *K*. *ozakii* (Shigematsu, 1953) Lin et al., 2007. To this number can be added two species formerly assigned to *Epiphyllum*, *K*. *shenzhenense* (Pan et al., 2010) comb. n. and *K*. *soliforme* (Fauré-Frémiet, 1908) comb. n. In addition, we are able to describe two new species of *Kentrophyllum* in the present paper, *K*. *bispinum* sp. n. and *K*. *strumosum* sp. n. (designated as *Epiphyllum* sp. in Fig 1).

Species of *Kentrophyllum* have been reported from marine and brackish-water habitats worldwide, some of them repeatedly, and this has led to some misidentifications and a certain amount of confusion (Table 1). *Kentrophyllum verrucosum* and *K*. *setigerum* are two common species that can be distinguished from one another by the presence (*K*. *verrucosum*) or absence (*K*. *setigerum*) of warts (clustered extrusomes) on the margin of the body [14, 16, 19, 22, 32, 35–37, 48]. Otherwise, their general morphology (e.g. body size and shape, number of macronuclear nodules, number and position of contractile vacuoles and the presence of spines) is closely similar.

**Table 1:** Morphological comparison of all known species of *Kentrophyllum*.

Species	LB (μm)^a^	Ma^c^	RSK/LSK^e^	n-CV^f^	P-CV^g^	Warts	PbP^l^	Spines	Biotope	Source
*K*. *antarcticum*	200–310	2	29–40/24–27	3–5	D^h^	Ab^j^	Ab	Pr	marine	[33]
*K*. *verrucosum*	125–250	3–7	38–45/28–33	4–7	D	Pr^k^	Pr	Pr	estuarine	Present work
	200	4	—	4	D	Pr	Ab	Pr	estuarine	[32]
	150	1^d^	—	5	D	Pr	Ab	Pr	marine	[36]
	200–250	3–6	—	7 ^d^	D	Pr	Pr	Pr	marine	[10]
	200–250	7^d^	—	7 ^d^	D	Pr	Ab^c^	Pr	marine	[11]
*K*. *setigerum*	80–350	4	—	3–4	D	Ab	—	Pr	marine	[35]
	100–140	5–14	—	5–6	D	Ab	Ab	Pr	marine	[19]
	130–300	4–9	36–40/30	5^d^	D	Ab	Pr	Pr	marine	[14]
	—^b^	6–8	35–40/—	1 or 2	D	Ab	Pr	Pr	estuarine	[48]
	200–250	4–9	—	5 ^d^	D	Ab	Pr	Pr	marine	[16]
	105	4	—	>1	D	Ab	—	Pr	marine	[49]
	100–350	4^d^	—	4–5^d^	D	Ab	Pr	Pr	marine	[11]
	100–140	5–14	—	5–6	D	Ab	—	Pr	marine	[19]
	90–200	4	—	several	D	Ab	—	Pr	—	[53]
	85–240	4	—	4	D	Ab	—	Pr	marine	[37]
	80–350	4	—	many	D	Ab	Pr	Pr	marine	[16]
	127–170	4	—	2 or 3	D	Ab	Ab	Pr	estuarine	[32]
*K*. *pseudosetigerum*	170	ca. 12	—	1	V^i^	Ab	Pr	Pr	marine	[12]
	175–200	many	—	1	—	Ab	Pr	Pr	marine	[38]
*K*. *raikovi*	360–500	11–14	—	many	V	Ab	Pr	Pr	marine	[14]
*K*. *ozakii*	170–230	17–20	—	1–3	V	Ab	—	Pr	estuarine	[50]
*K*. *fibrillatum*	90	2	24/20–22	1	D	Ab	Pr	Pr	marine	[12]
*K*. *soliforme* (Fauré-Fremiet, 1908) comb. n.	90	4	—	1	D	Ab	Pr	Ab	estuarine	[51]
*K*. *hohuensis* (Wang & Nie, 1933) comb. n.	200–280	4	—	4–10	D	Ab	—	Ab	freshwater	[52]
*K*. *shenzhenense* (Pan et al., 2010) comb. n.	120–180	4	20-29/10-26	4	D	Ab	—	Ab	estuarine	[27]
*K*. *bispinum* sp. n.	120–290	2–6	35–45/26–35	9–14	V, D	Ab	Ab	Pr	estuarine	Present paper
*K*. *strumosum* sp. n.	125–240	4–9	41-55/35-44	4–7	D	Pr	Ab	Ab	estuarine	Present paper
*K*. *qingdaoense* sp. n.	90–250	2–9	30–48/35–53	6–10	V, D	Ab	Ab	Pr	estuarine	[22]

^a^ Length of body in living cells;

^b^ Data not given;

^c^ Number of macronuclei;

^d^ Data based on illustration;

^e^ Number of right somatic kineties/number of left somatic kineties;

^f^ Number of contractile vacuoles;

^g^ Position of contractile vacuoles;

^h^ ventral;

^i^ dorsal;

^j^ absent;

^k^ present;

^l^ Peribuccal papillae;

—, N/A

*Kentrophyllum setigerum* was originally described by Quennerstedt [36] who characterized it mainly by the following characters: body measuring 80–350 μm in length, with 3–4 contractile vacuoles lying in dorsal half, 4 macronuclear nodules, and spines along the margin of the cell. There were no warts on the margin of the cell [35]. Kahl [10] considered *Litosolenus armatus* Stokes, 1893 to be a synonym *L*. *setigerum*, and we agree with him although the spines were shown by Stokes [32] as occurring along the oral slit, which is not the case in any of the known species of *Kentrophyllum*. However, this is probably an error of description on the part of Stokes. Ozaki & Yagiu [19] described *Loxophyllum multinucleatum* from Japan, and their species was identified as *Kentrophyllum verrucosum* by Lin et al. [22]. However, the extrusomes of this species are evenly distributed along the body margin and not clustered to form warts; therefore, we have identified it as *K*. *setigerum* because it also corresponds to the diagnosis of the species in regard to the rest of its morphology.

*Kentrophyllum verrucosum* was originally described as *Litosolenus verrucosus* by Stokes [32] from specimens collected in a marsh. Key diagnostic features of this species were overlooked by some subsequent investigators, which resulted in several misidentifications. We have concluded that records of *K*. *verrucosum* reported by Dragesco [14], Czapik & Jordan [48], and Carey [16] actually should be attributed to *K*. *setigerum* because they do not mention or illustrate the warts (papilliform elevations clearly described by Stokes [32]) associated with spines that are diagnostic for *K*. *verrucosum* [10, 11]. The record of *K*. *setigerum* in Sauerbrey [36], in which extrusomes are described as clustered in groups (warts) also can be identified as *K*. *verrucosum*. The ciliate identified as *K*. *verrucosum* by Lin et al. [22] fits the description of the species by having no warts and having single spines distributed evenly around the margin of the cell; however, some of its other characteristics differ from those of *K*. *verrucosum* and all other known species of *Kentrophyllum*. Therefore, we redescribe it in the present paper as *Kentrophyllum qingdaoense* sp. n.

*Loxophyllum hohuensis* Wang & Nie, 1933 appears to represent another member of *Kentrophyllum*, judging by the morphology of the living cell (e.g., laterally compressed body, four macronuclear nodules, many CVs in the dorsal half of the cell, extrusomes evenly distributed along the entire margin of the body except for the oral slit, absence of spines. Although Wang & Nie [49] did not present a detailed description of its infraciliature their illustration shows a general pattern of ciliature that is typical for species of *Kentrophyllum*. Therefore, we propose a new combination, *Kentrophyllum hohuensis* (Wang & Nie, 1933) comb. n. [basionym *Loxophyllum hohuensis* Wang & Nie, 1933].

**Fig 2:**
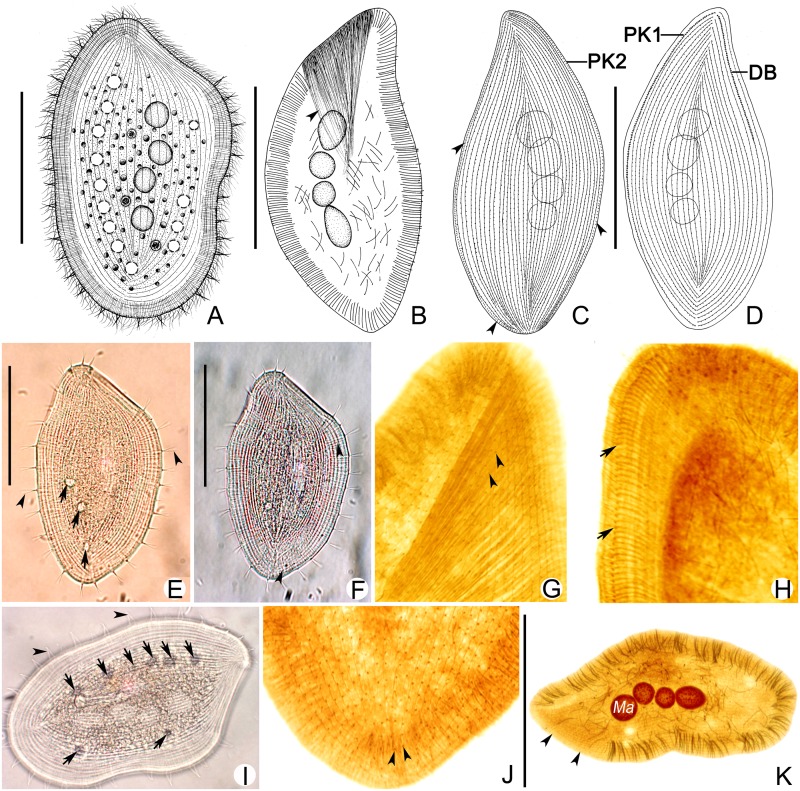
*Kentrophyllum bispinum* sp. n.; living individual (A-D, E, F, I) and cells stained with protargol (G, H, J, K). **A.** Right lateral view of representative specimen. **B.** Distribution of extrusomes and developed nematodesmata (arrowhead). **C, D.** Ciliary patterns of right (C) and left (D) side; arrowheads, perioral kinety 2. **E, I.** Right lateral view. Arrows, contractile vacuoles; arrowheads, double-spines. **F.** Right lateral view, showing the extrusomes (arrowheads). **G.** Anterior part of right side, showing the anterior suture (arrowheads). **H.** Anterior part of right side; arrows indicate the perioral kinety 1. **J.** Posterior part of left side showing the posterior suture (arrowheads). **K.** Distribution of extrusomes; arrowheads, oral slit. Ma, macronuclear nodules; DB, dorsal brush; PK1, perioral kinety 1; PK2, perioral kinety 2. Scale bars, 100 μm.

***Kentrophyllum bispinum* sp. n.** urn:lsid:zoobank.org:act: 9DA4C4DE-0E71-47DB-8E2D-BC4097F205A7 (Fig 2; Table 2)

**Table 2:** Morphometric data^a^ of *Kentrophyllum verrucosum* (1st line), *K*. *bispinum* n. sp. (2nd line) and *K*. *strumosum* n. sp. (3rd line).

Characters	Range	Mean	SD	CV	n
length of body	100–210	156.0	± 30.58	19.6	27
	120–290	199.1	± 40.50	20.3	21
	125–240	178.9	± 31.34	17.5	19
width of body	83–125	101.8	± 11.88	11.7	27
	60–130	91.1	± 16.74	18.4	21
	55–120	83.7	± 18.32	21.9	19
number of RSK^b^	38–45	40.8	± 1.71	4.2	24
	35–45	38.5	± 2.56	6.7	20
	41–55	46.5	± 4.03	8.7	19
number of LSK^c^	28–33	30.5	± 1.30	4.3	22
	26–35	30.4	± 2.39	7.9	16
	35–44	38.2	± 2.46	6.4	17
number of Ma^d^	3–7	4.2	± 0.86	20.5	27
	2–6	3.9	± 1.06	27.2	21
	4–9	6.2	± 1.32	21.3	19
length of Ma	13–30	19.3	± 4.85	25.1	24
	12–35	20.2	± 5.51	27.3	20
	10–30	16.9	± 5.13	30.4	19
width of Ma	12–25	16.0	± 2.75	17.1	24
	12–27	15.7	± 3.91	25.0	20
	10–20	12.3	± 3.51	28.5	19
length of extrusomes	14–18	16.0	± 0.73	4.6	25
	15–15	0	0	0	20
	6–20	12.1	± 3.87	32.1	10
number of warts	6–19	10.2	± 2.99	29.4	23
	—^e^	—	—	—	—
	7–15	11.6	± 2.3	19.8	18

^a^ All measurements in μm. Data based on specimens stained with protargol; CV, coefficient of variation expressed as a percentage; SD, standard deviation; n, sample size.

^b^ Number of right somatic kineties; perioral kinety 2 included.

^c^ Number of right somatic kineties; perioral kinety 1 and dorsal brush kinety included.

^d^ Macronuclear nodules.

^e^ N/A.

**Diagnosis.** Leaf-shaped body measuring 120–350 μm × 60–180 μm in vivo; with 28–35 double-spines along cell margin excluding the buccal area, 2–6 macronuclear nodules, 9–15 contractile vacuoles along both ventral and dorsal margins, 35–45 right kineties, and 26–35 left kineties. Extrusomes evenly distributed along body margins excluding buccal area.

**Type locality and features of the habitat.** Collected 24 October 2012 from the intertidal zone near Haibin Park, Zhanjiang, Guangdong Province, China (21° 11’ N; 110° 18’ E). Water temperature 26.0°C, salinity 25‰, pH 7.8.

**Deposition of type slides.** A holotype slide (protargol preparation with the holotype specimen marked in circle) was deposited in the collection of the Laboratory of Protozoology, Ocean University of China (OUC), Qingdao, China, with registry number WL20121024-01. One paratype slide (protargol preparation with paratype specimens marked) was deposited in the collection of the Natural History Museum, London, UK, with registry number NHM2014.10.7.1.

**Etymology.** The specific epithet is formed from the Latin prefix *bi* (two, double) and the noun *spin* ([n]; thorn, sting) and refers to the paired double-spines on the margin of the cell.

**Description.** Size of cells highly variable, ranging from 120–350 μm × 60–180 μm in vivo. Body shape varying from slender and leaf-shaped to nearly globular when in different modes of movement, without anterior end narrowed to form a “neck” region typical of many haptorians (Fig 2E and 2F). Margin of cell consists of well-defined hyaline fringe, approximately 15 μm in width. Length to width ratio of body 4–5:1 when fully extended; central region of body hunches on left side when cell contracts (Fig 2A). Two to 6 ellipsoidal macronuclear nodules, each measuring 12–35 μm × 12–27 μm in vivo, located near ventral region of cell, usually detectable in living cells (Fig 2I). Micronucleus not observed. Contractile vacuoles numbering 9–15 and measuring 3–10 μm in diameter, with 3–5 located in ventral half of cell and 6–10 in dorsal half (Fig 2A, 2E and 2I). Extrusomes slender and bar-shaped, straight to slightly curved, approximately 15 μm long, densely arranged and evenly distributed along entire margin of body excluding oral area, some scattered in cytoplasm (Fig 2B and 2F). Pellicle thin, without cortical granules. Right side flat and densely ciliated; cilia 4–6 μm long. Left side also densely ciliated, with many conspicuous, shallow, longitudinal grooves, and slightly arched owing to presence of food vacuoles. Twenty-eight to 35 double-spines distributed evenly around entire margin of cell except for region of cytostome; slender, stiff, and immobile in vivo, with slightly overlapped tips, interdigitated or dispersed when in different focal planes (Fig 2A, 2E, 2F and 2I). Movement by left side of body slow gliding on substrate, rarely swimming in water column, often stationary.

Ciliary pattern as shown in Fig 2C, 2D, 2H and 2J. Two perioral kineties (PK1, 2) around cytostome; PK1 on left side of oral slit, formed of closely spaced dikinetids in anterior 2/3, continuing as a row of closely spaced monokinetids; PK2 on right of oral slit, composed of more closely spaced dikinetids, making a complete circuit around margin of cell. Right side of body with 35–45 closely spaced kineties, with anterior parts of medial kineties shortened to form a distinct anterior suture (Fig 2C). Left side of body with 26–35 kineties, which together with PK1 and dorsal brush kinety form conspicuous anterior and posterior medial sutures (Fig 2D). Dorsal brush kinety (DB) extending to nearly half length of cell and composed of regularly spaced dikinetids (Fig 2D). Nematodesmata well-developed, all originating from kinetosomes of perioral kinety and extending into the cytoplasm (Fig 2B and 2G).

**Comparison of *Kentrophyllum bispinum* sp. n. with congeners.** *Kentrophyllum bispinum* sp. n. is the only species in its genus with paired spines on the margin of the cell [12, 14, 22, 33, 50] (Table 1).

**Fig 3:**
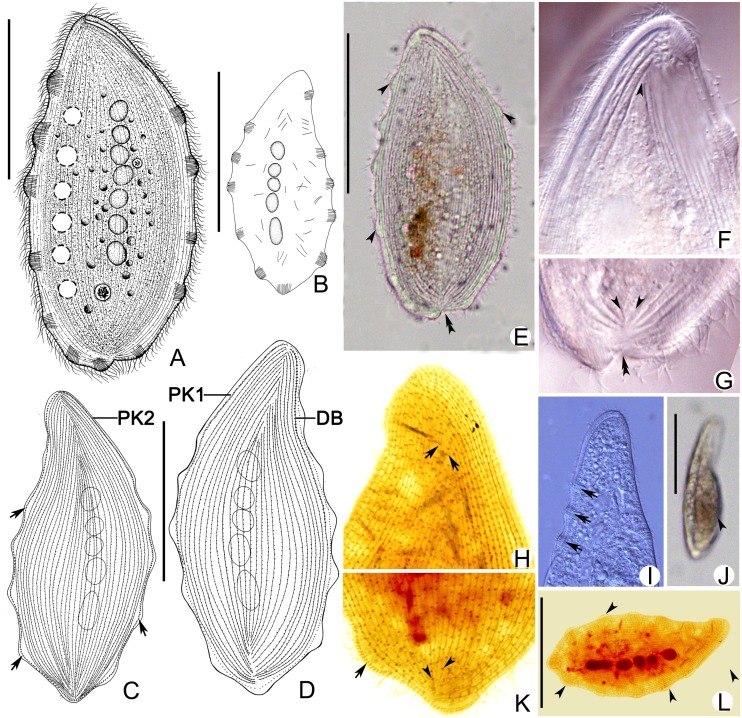
*Kentrophyllum strumosum* sp. n.; living individual (A-D, E-G, I, J) and cells stained with protargol (H, K, L). **A.** Right lateral view of representative specimen. **B.** Left lateral view, showing distribution of extrusomes and warts (arrowheads). **C, D.** Ciliary patterns of right (C) and left (D) side; arrows, perioral kinety 2. **E.** Right lateral view, showing erose posterior (double arrowheads) and warts (arrowheads). **F, I.** Anterior part of left side showing extrusomes in the warts and suture (arrowhead). **G.** Posterior part of left side showing grooves (arrowheads) and erose posterior (double arrowheads). **H.** Anterior part of right side indicating suture (arrows). **J.** Lateral side view, showing the arched left side (arrowhead). **K.** Posterior part of left side, showing suture (arrowheads). **L.** Right lateral view, showing perioral kinety 2 (arrowheads). Ma, macronuclear nodules. DB, dorsal brush; PK1, perioral kinety 1; PK2, perioral kinety 2. Scale bars, 100 μm.

***Kentrophyllum strumosum* sp. n.** urn:lsid:zoobank.org:act: 234BC0E6-DC24-46D1-BE7A-53FE547FD79D (Fig 3; Table 2)

**Diagnosis.** Leaf-shaped body measuring 120–235 μm × 60–130 μm in vivo; with 4–9 macronuclear nodules, 4–7 contractile vacuoles along dorsal margin, 41–55 right kineties, and 35–44 left kineties. Extrusomes grouped into warts around the margin of the body except for buccal area.

**Type locality and features of the habitat.** Collected (28 November 2011) from a mangrove wetland, in Huizhou, Guangdong Province, China (22° 44’ N; 114° 31’ E). Water temperature 23.5°C, salinity 11‰, pH 7.4.

**Deposition of type slides.** A holotype slide (protargol preparation with the holotype specimen marked in circle) was deposited in the collection of the Laboratory of Protozoology, Ocean University of China (OUC), Qingdao, China, with registry number WL20111128-02. One paratype slide (protargol preparation with paratype specimens marked) was deposited in the collection of the Natural History Museum, London, UK, with registry number NHM2014.10.7.2.

**Etymology.** The specific epithet is formed from the Latin adjective *strumos us*,-a,-um ([m, f, n]; warts) and refers to the warts on the margin of the cell.

**Description.** Size of cells highly variable, ranging from 120–235 μm × 60–130 μm in vivo. Body shape varying from broadly leaf-shaped when swimming to nearly round when stationary, slightly contractile, without anterior end narrowed to form a “neck” region typical of many haptorians, posterior portion of cell on left side often sunken in (Fig 3E and 3G). Length to width ratio of body 3–4:1 (Fig 3E). Margin of cell consists of flat, thin, hyaline fringe approximately 10 μm in width. Four to 9 ellipsoid macronuclear nodules, each measuring approximately 12 μm × 10 μm in vivo, located in central part of body. Micronucleus not observed. Contractile vacuoles numbering 4–7 and measuring 5–12 μm in diameter, located near dorsal margin of cell. Extrusomes bar-shaped, approximately 15 μm long, clustered to form 7–15 warts distributed at wide intervals in hyaline fringe excluding buccal area (Fig 3E, 3F and 3I). Pellicle thin, without cortical granules. Cytoplasm grayish to dark in central region of cell owing to presence of food vacuoles. Right side flat and densely ciliated; cilia ~8 μm long. Left side distinctly arched when cell is swimming (Fig 3E), with many conspicuous, shallow, longitudinal grooves, and forming conspicuous sutures on both anterior and posterior part (Fig 3F and 3G). Movement by left side of body slow gliding on substrate or occasionally swimming in water, often stationary.

Ciliary pattern as shown in Fig 3C, 3D, 3H and 3K. Two perioral kineties (PK1, 2) around cytostome: PK1, left of oral slit, formed of closely spaced dikinetids in its anterior 2/3, continuing as a row of monokinetids (Fig 3D); PK2, on right of oral slit, forming a closed loop, composed of closely spaced dikinetids making a complete circuit around margin of cell (Fig 3C). Right side of body with 41–55 kineties, including PK2, forming a consipicuous anterior suture that extends about 1/3 length of body and an inconspicuous, subterminal posterior suture (Fig 3C and 3K). Left side with 35–44 kineties that form distinct sutures in both anterior and posterior regions (Fig 3D). Dorsal brush (DB) extending to half length of cell as row of densely spaced dikinetids (Fig 3D).

**Comparison of *Kentrophyllum strumosum* sp. n. with congeners.** This species is the only member of its genus with extrusomes clustered into warts that have no spines associated with them [22, 27, 32, 49, 51, 52].

#### *Kentrophyllum setigerum* (Quennerstedt, 1867) Petz et al., 1995

**Synonymy.** *Loxophyllum setigerum* in Quennerstedt, 1867, p. 6 (original description); *Litosolenus armatus* in Stokes, 1893, p. 301 (description of American population); *Loxophyllum multinucleatum* in Ozaki & Yagiu, 1943, p. 41 (description of Japanese population); *Loxophyllum verrucosum* in Dragesco, 1965, p. 373 (description of African population); *Loxophyllum verrucosum* in Czapik & Jordan, 1976, p. 435 (description of Baltic population); *Loxophyllum verrucosum* in Carey, 1992, p. 74 (taxonomic key); *Kentrophyllum setigerum* in Petz et al., 1995, p. 55 (new combination).

**Emended diagnosis (based on all known descriptions).** Body measuring 80–350 μm long in vivo, with 4–14 macronuclear nodules, several contractile vacuoles along dorsal margin, 35–40 right and ~30 left somatic kineties; extrusomes evenly distributed along margin of body excluding oral region; spines present; peribuccal papillae absent or present; Brackish or marine habitat.

#### *Kentrophyllum verrucosum* (Stokes, 1893) Petz et al., 1995 (Fig 4; Table 2)

**Fig 4:**
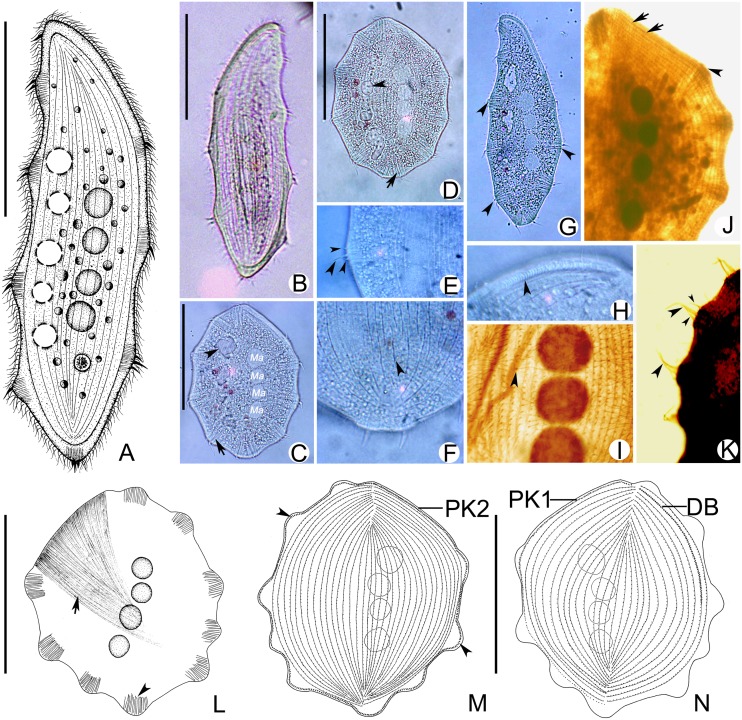
*Kentrophyllum verrucosum*; living individuals (A–H, L–N) and cells stained with protargol (I-K). **A.** Right lateral view of representative specimen. **B.** Left lateral view of representative specimen. **C, D.** Right lateral views of contracted specimens; arrowheads, contractile vacuoles; arrows, extrusomes. **E, K.** Margin of cell under higher magnification; arrowheads, spines. **F.** Posterior part of left side, showing grooves (arrowheads). **G.** Right lateral view showing warts (arrowheads). **H.** Anterior part of left side showing peribuccal papillae (arrowheads). **I.** Middle part of right side showing somatic kineties (arrowheads). **J.** Right lateral view. Arrows, perioral kinety 2; arrowheads, warts. **L.** Distribution (arrowhead) and development (arrow) of nematodesmata. **M, N.** Ciliary patterns of right (M) and left (N) sides; arrows, perioral kinety 1. Ma, macronuclear nodules. DB, dorsal brush; PK1, perioral kinety 1; PK2, perioral kinety 2. Scale bars, 100 μm.

**Synonymy.** *Litosolenus verrucosus* in Stokes, 1893, p. 302 (original description), *Loxophyllum setigerum* in Sauerbrey, 1928, p. 369 (misidentification), *Loxophyllum verrucosum* in Kahl, 1931, p. 201 (new combination), *Loxophyllum verrucosum* in Kahl, 1933, p. 63 (description of German population), *Kentrophyllum verrucosum* in Petz et al., 1995, p. 55 (new combination).

**Emended diagnosis (based on all known descriptions and new data from the present study).** Body measuring 125–250 μm × 65–185 μm in vivo, with 3–7 macronuclear nodules, 4–7 contractile vacuoles along the dorsal margin, 38–45 right and 28–33 left kineties. Extrusomes clustered into warts distributed along margin of body excluding the oral area; each wart with one or more soft spines. Peribuccal papillae present or absent. Brackish or marine habitat.

**Deposition of voucher material.** One voucher slide (protargol preparation) was deposited in the collection of the Laboratory of Protozoology, Ocean University of China (OUC), Qingdao, China, with registry number WL20121112-03.

**Features of the habitat.** Sample collected on12 November, 2012 from a mangrove wetland on Techeng Island, Zhanjiang, Guangdong Prov., China (21° 14’ N; 110° 23’ E). Water temperature 22.4°C, salinity 25‰, pH 7.8.

**Description of population from Zhanjiang.** Cells highly variable in size, measuring 125–250 μm × 65–185 μm in vivo, usually 170–200 μm × 90–150 μm. Body ranging in shape from slender when swimming to nearly oval when stationary (Fig 4B–4D and 4G). Length to width ratio 3–4:1. Margin of cell flat, thin, hyaline, ~10 μm wide (Fig 4B). Three to 7 (usually 4) macronuclear nodules located near center of cell; nodules spherical to ellipsoid, measuring 10–15 μm × 10–12 μm in vivo (Fig 4C, 4D and 4G). Micronucleus not observed. Four to 7 contractile vacuoles, with diameter of 5–12 μm, located in dorsal half of cell (Fig 4C, 4D and 4G). Extrusomes bar-shaped, ~15 μm long, always clustered to form 6–19 warts along margin of body excluding oral area; interval between warts usually concave. Each wart with 1–4 (usually 3) soft spines, each spine thick at base and tapering to sharp point; one spine in each cluster always much longer (6–7.5 μm) than others (3–4.5 μm) (Fig 4A, 4C, 4E, 4G and 4K). Pellicle thin; cytoplasm gray to dark in center of cell, depending on number of food vacuoules present, and often containing numerous, refringent globules measuring 2–5 μm in diameter. Right side flat with many inconspicuous, longitudinal, shallow grooves (Fig 4G); left side slightly to distinctly arched, with many conspicuous, longitudinal, shallow grooves (Fig 4F). Thirty to 50 peribuccal papillae densely arranged along left edge of oral slit (Fig 4H). Movement by left side of body slow gliding on substrate, usually stationary, rarely swimming in water column.

Ciliary pattern as shown in Fig 4M, 4N and 4J. Two perioral kineties (PK_1, 2_): PK_1_, lying along left side of oral slit, formed of regularly spaced dikinetids in anterior 1/2 of body and continuing toward posterior as row of monokinetids; PK_2_ (peripheral kinety) consists of closely spaced dikinetids and surrounds entire right margin of body. Right-hand somatic kineties densely ciliated, numbering 38–45 in (including PK_2_) and forming a distinct anterior suture and an inconspicuous posterior suture. Left side of body also densely ciliated, with 28–33 somatic kineties (including PK1 and dorsal brush kinety) forming a distinct suture at both anterior and posterior ends of the body. Dorsal brush kinety extending half of body length, formed of densely spaced dikinetids. Nematodesmata well-developed, up to 150 μm long.

**Remarks.** The peribuccal papillae present on the ciliates in our sample from southern China were not mentioned in the original description [32] and in Sauerbrey [36]; however, this characteristic was observed by Kahl [10, 11]. Therefore, the peribuccal papillae were probably overlooked by Stokes [32] and Sauerbrey [36] or they may be present on some populations but not others.

**Fig 5:**
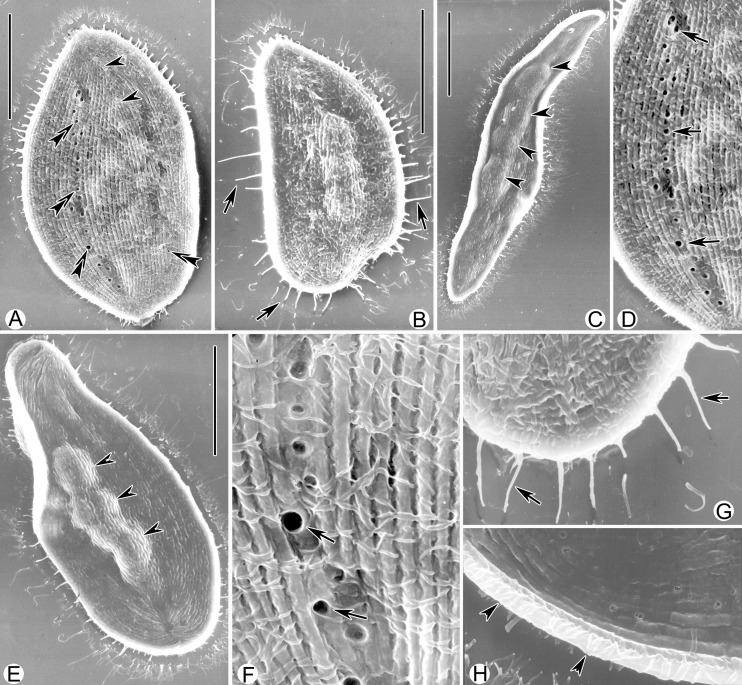
*Kentrophyllum qingdaoense* sp. n.; photomicrographs of SEM (A–H). **A, B.** Right lateral views. Arrowheads, suture; arrows, single spine; double-headed arrows, pores of contractile vacuoles. **C, E.** Left lateral views. Arrowheads, macronuclear nodules. **D, F.** Right lateral views, indicating the pores of contractile vacuoles (arrows). **G.** Posterior part of right side, showing single spines (arrows). **H.** Body margin (arrowheads). Scale bars, 50 μm.

***Kentrophyllum qingdaoense* sp. n.** urn:lsid:zoobank.org:act:AC99A4BF-56D1-4B68-AFED-3956112D2C0B (Fig 5; Table 1)

**Synonymy.** *Kentrophyllum verrucosum* sensu Lin et al., 2005, p. 130 (description of Chinese population, misidentification).

**Diagnosis.** Leaf-shaped body, measuring 90–250 μm × 60–160 μm in vivo; with 30–48 right and 35–53 left somatic kineties, 6–8 contractile vacuoles along both ventral and dorsal margins, and 2–9 macronuclear nodules. Two types of extrusomes; type 1 mostly distributed regularly along ciliary rows, with some scattered in cytoplasm, and type 2 evenly distributed along margins of body excluding oral area. Peribuccal papillae absent.

**Etymology.** The species is named after the sampling site where it was discovered.

**Type locality, features of the habitat, deposition of type slides and description.** See Lin et al. [22].

**Description based on SEM observation.** Body shape varying from slender and leaf-shaped to oval-shaped (Fig 5A–5C), no typical “neck” region like that of many pleurostomatids. Single spine projecting from margin of cell excluding oral slit (Fig 5B, 5E and 5G). Ellipsoidal macronuclear nodules located central region of cell (Fig 5C and 5E). Pores of contractile vacuoles distributed in rows mostly near dorsal margin and seldom near ventral margin (Fig 5A, 5D and 5F). Right side flat and densely ciliated, with many conspicuous, shallow, longitudinal grooves (Fig 5A, 5B, 5D and 5F) while left side sparely ciliated (Fig 5C, 5E and 5H).

**Comparison of *Kentrophyllum qingdaoense* sp. n. with congeners.** Seven of the known species of *Kentrophyllum* resemble *K*. *qingdaoense* in having single spines; however, *K*. *qingdaoense* differs from them as follows [12, 14, 22, 33, 50]:

- *K*. *antarcticum* by has fewer (24–27 vs.35–53) somatic kineties on the left side and fewer (3–5 vs. 6–10) contractile vacuoles
- *K*. *setigerum* has fewer (~30 vs. 35–53) somatic kineties on the left side and contractile vacuoles in a different position (dorsal vs. dorsal and ventral)
- *K*. *pseudosetigerum* has more (~12 vs. 2–9) macronuclear nodules and far fewer (1 vs. 6–10) contractile vacuoles
- *K*. *fibrillatum* has fewer somatic kineties (24 vs. 30–48 on right side and 20–22 vs. 35–53 on left side)
- *K*. *raikovi* has a significantly larger body (360–500 vs. 90–250 μm long) and more (11–14 vs. 2–9) macronuclear nodules
- *K*. *armatum* has fewer (3–4 vs. 6–10) contractile vacuoles in a different position (dorsal vs. dorsal and ventral)
- *K*. *ozakii* has many more macronuclear nodules (170–230 vs. 2–9).

### Key to all known species of *Kentrophyllum*

1. With spines along body margin……………………. . .…. . . .. . . .…….…. . .……. . .. . . 2

Without spines along body margin…………………………. . .. . ..…..…………….. 10

2. With single-spine along body margin ……………. . . .. . . .. . . .. . . .…….…….………. 4

With double-spines or soft spine along body margin…….………..…. . . .………. 3

3. With warts along body margin………………..……………..……. . . .. *K*. *verrucosum*

No wart along body margin………………….……………..…….……. *K*. *bispinum*

4. The contractile vacuoles along both dorsal and ventral margins…. . . .. *K*. *qingdaoense*

The contractile vacuoles along dorsal or ventral margin……….….………………. 5

5. The contractile vacuoles along dorsal margin……………….…………….…… . 6

The contractile vacuoles along ventral margin………………..………….……. . .. . .. 7

6. With two macronucleus ……………………………………..……………..…. . . . 8

With more than two macronuclei……………………….……. . .……. . . .. *K*. *setigerum*

7. With one contractile vacuole …………………………. . . .….…. . . *K*. *pseudosetigerum*

With many contractile vacuoles………………….….………….….……………. . . . 9

8. With one contractile vacuole. . . .. . . .. . . .. . . .. . . .. . . .. . . .. . . .. . . .. . . .. . . .. . . .. . . .. . . .. . .. . .. *K*. *fribrillatum*

With several contractile vacuoles ……………………. . .……………. . . . *K*. *antarcticum*

9. With less 15 macronuclei……….……………..……………………. *K*. *raikovi*

More than 15 macronuclei……….…………………………..………. . . . *K*. *ozakii*

10. With warts along body margin………………………..…………. . . . *K*. *strumosum*

No wart along body margin……………….……………….…………….……. . .. . . 11

11. With one contractile vacuole …………………………. . .…….…… *K*. *soliforme*

With several contractile vacuoles…………………………………….…….…. . .. . . 12

12. The body length more than 200 μm…………………………..…. . . . *K*. *shenzhenense*

The body length less 200 μm…………………….………..…………. . . *K*. *hohuensis*
